# Identification of Key Factors Governing Compressive Strength in Cement-Stabilized Rammed Earth: A Controlled Assessment of Soil Powdering Prior to Mixing

**DOI:** 10.3390/ma19010088

**Published:** 2025-12-25

**Authors:** Piotr Narloch, Łukasz Rosicki

**Affiliations:** Faculty of Civil Engineering, Warsaw University of Technology, Al. Armii Ludowej 16, 00-637 Warsaw, Poland

**Keywords:** rammed earth, cement-stabilized, compressive strength, soil powdering, moisture content, cement content, curing time, loading direction, bulk density

## Abstract

This study evaluated the influence of soil preparation method and initial moisture content on the compressive strength of cement-stabilized rammed earth (CSRE). Cube samples stabilized with 7–12% cement were compacted using a manual rammer, cured for up to 28 days, and tested according to adapted EN 12390-3 procedures. These results indicated that eliminating the powdering step improved laboratory efficiency and produced specimens more representative of field practice. These findings demonstrate that labor-intensive powdering of natural soils is unnecessary, provided that moisture is accurately determined, thereby improving both laboratory efficiency and consistency with field practice. The outcomes contribute to optimizing laboratory methodologies for earthen construction materials.

## 1. Introduction

### 1.1. Research Background

Rammed earth is an increasingly recognized construction material in the context of sustainable building, valued for its low embodied energy, local availability, and favorable thermal mass properties. Despite its long history of use, the mechanical characterization of rammed earth—particularly its compressive strength—remains a significant research challenge due to the inherent variability of the material and its sensitivity to preparation methods.

One of the main obstacles in standardizing mechanical testing lies in the absence of uniform sample preparation procedures. In particular, the influence of the mixing technique—whether the soil component is used in a moist, cloddy form typical of on-site construction or as a dry, powdered soil commonly used in laboratory studies—has not been sufficiently investigated [1,2]. Additionally, moisture content, widely recognized as a critical factor influencing compaction and strength development, often exhibits considerable variation both in experimental work and in practical applications [3]. Even small differences in the initial moisture content can lead to significant deviations in compressive strength results. Earlier studies also demonstrated that the mineral composition of soil significantly affects the compressive strength of CSRE, underlining the importance of controlling the source material [4].

The aim of this study is to systematically assess the effect of two different mixture preparation techniques and varying initial moisture content on the compressive strength of rammed earth specimens made from the same clay-rich soil. The central research question addresses whether soil powdering (powdered soil) produces different UCS outcomes compared to natural clods (natural soil), while other factors such as cement content, curing time, and loading direction are included as control and contextual variables to validate the robustness of the findings. The first technique reflects field-oriented practice using pre-moistened cloddy soil, while the second follows a laboratory-standard approach based on dry mixing. By isolating the influence of these variables, the study seeks to provide empirically grounded recommendations for improving the comparability of laboratory results with real-world construction conditions. The findings have important implications for both methodological advancement in rammed earth research and the practical application of earthen materials in modern sustainable construction [5].

Unconfined compressive strength (UCS) testing, particularly in the context of cement-stabilized rammed earth, is inherently demanding in terms of material preparation, precision, and time investment. Each specimen must be carefully mixed, compacted, demolded, cured under controlled conditions, and subsequently tested with appropriate alignment and load application protocols. Given the labor-intensive nature of the procedure and the curing periods involved—often extending over 28 days—the number of specimens was intentionally limited. The objective was to maintain a manageable and focused experimental scope, prioritizing the quality, consistency, and comparability of the results.

### 1.2. Construction Method

**Figure 1:**
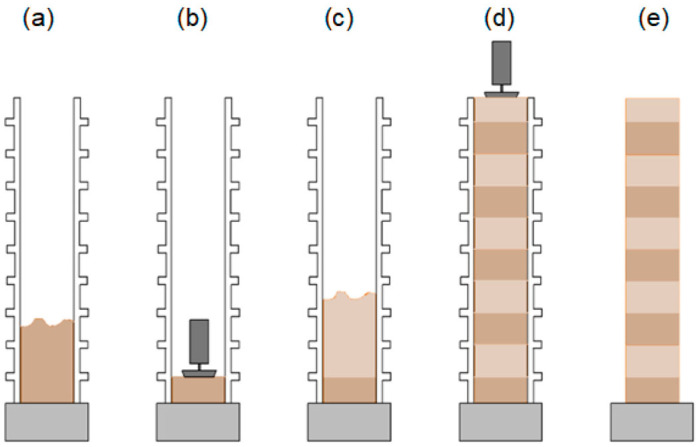
Schematic representation of the rammed earth wall construction process using the CSRE monolithic wall system. The CSRE system utilized continuous monolithic compaction, steel guiding frames, and sequential layer deposition to control density and wall uniformity. Distinct construction layers are indicated by different colors. The process includes the following stages: (**a**) Erection of formwork and placement of the first layer of a moist soil–cement mixture; (**b**) Compaction of the placed layer; (**c**) Addition of the subsequent moist soil–cement layer; (**d**) Repetition of layering and compaction to build up the wall structure; (**e**) Removal of formwork, revealing the continuous monolithic CSRE (Cement-Stabilized Rammed Earth) wall [10].

The rammed earth construction technique involves the layered compaction of a moist earth mixture placed within formwork set on a stable foundation [6,7] (Figure 1). The primary component of the mixture is inorganic mineral soil extracted from beneath the humus layer, typically sourced directly from the construction site. Depending on the grain size distribution of the natural soil, the mixture is modified by adding sand, gravel, clay-rich materials, and a stabilizing agent [8].

**Figure 2:**
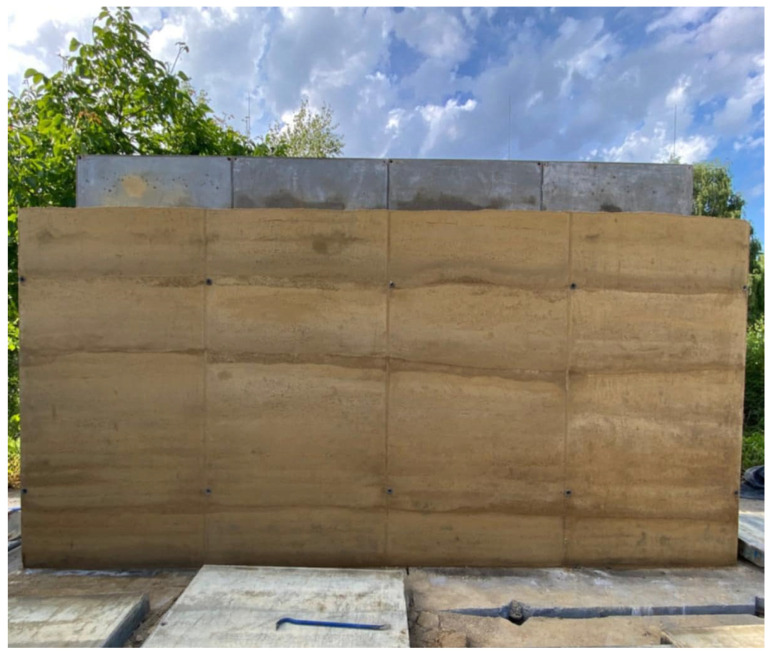
Cement-stabilized rammed earth wall immediately after formwork removal. Layer discontinuities were inherent to the stratified compaction process and could be minimized by maintaining uniform moisture content and consistent compaction energy.

These components are initially combined in an air-dry state, after which water is added to achieve the moisture content necessary for effective compaction [9]. The resulting loose, moist mixture is placed in the formwork in successive layers and compacted using either a manual or mechanized rammer. Once one layer is adequately compacted, the next is added, and the process is repeated until the intended height of the wall element is reached. Finally, the formwork is removed. An example of a cement-stabilized rammed earth wall from Poland, located in a temperate climate, is shown in Figure 2.

The purpose of layer-by-layer compaction is to reduce the porosity of the material, thereby increasing its mechanical strength. The load-bearing capacity and long-term durability of the finished wall depend on the composition of the earth mixture and the type and quantity of stabilizing additives used.

### 1.3. Empirical Field Method for Estimating Suitable Moisture Content

Determining the optimal moisture content using the Proctor compaction method is generally impractical on construction sites due to equipment and time requirements. The issue of establishing a suitable moisture content for rammed earth mixtures under field conditions is addressed in the New Zealand standard NZS 4298:1998 [11] which describes a simple and rapid empirical technique known as the drop-ball test.

**Figure 3:**
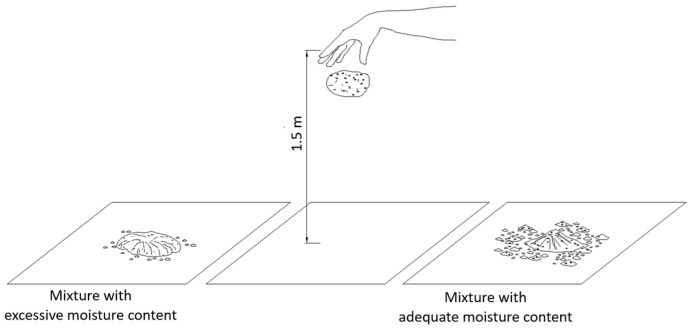
Schematic representation of the field assessment of rammed earth mixture moisture content using the drop-ball method, in accordance with NZS 4298:1998 [11].

This method involves manually forming a ball from the moist soil mixture and dropping it from a height of 1.5 m onto a hard, flat surface (Figure 3). The disintegration pattern of the ball upon impact serves as an indicator of whether the moisture content is appropriate for constructing vertical rammed earth elements. If the ball breaks into several large fragments, the moisture content is considered suitable. If it shatters into small particles, the mixture is too dry; if it remains intact, the moisture content is too high [12].

It must be emphasized, however, that this method is qualitative and does not provide a precise determination of the optimal moisture content. Due to its subjective interpretation and lack of quantifiable assessment criteria, it should be regarded solely as a supplementary on-site control procedure. It is not appropriate for use as a reference method in laboratory testing or for research requiring accurate moisture calibration. Alternative field procedures such as miniature Proctor compaction or slump-type cone tests could also be applied.

### 1.4. Compressive Strength Studies

As a result of the conducted research, a tool based on artificial neural networks (ANN) was developed to accurately predict the compressive strength of cement-stabilized rammed earth (CSRE) mixtures using basic soil parameters such as grain size distribution and moisture content [10]. The predictions generated by the neural network, implemented in Statistica software (version 14.0.1), demonstrated high accuracy, with a mean absolute error (MAE) of 0.565% relative to the target moisture content. Notably, the prediction error was lower than the variability typically encountered during CSRE mixture preparation under on-site construction conditions, which are often influenced by fluctuating environmental factors.

It is important to emphasize that, despite the availability of this predictive tool, a scientifically justified and practical approach still involves direct verification of compressive strength [13]. This can be accomplished through a relatively simple procedure involving the collection of in situ soil, its modification, and the preparation of a series of test specimens. Such an approach accounts for local soil variability and field conditions and provides a valuable complement to model-based predictions.

It should also be noted that many scientific publications concerning rammed earth employ laboratory methods based on synthetic mixtures composed of clay, silt, sand, and gravel with different stabilizers prepared in various ways (Table 1). These mixtures do not fully reflect the properties of natural soils, which often contain natural clay and silt fractions and tend to occur in the form of compacted or aggregated structures. This has a significant impact on their rheological behavior, workability, and compactability, all of which influence the final mechanical performance of the material. Therefore, laboratory procedures should account for these discrepancies when interpreting results and translating them to real-world applications.

Furthermore, although several guidelines and standards—both in the literature and in emerging codes of practice—propose recommended moisture ranges for soils used in rammed earth construction, they do not provide a precise or universally applicable method for determining the optimal moisture content in field conditions [14]. In practice, moisture content is highly variable and influenced by environmental factors such as temperature, solar radiation, ambient humidity, and precipitation. Accordingly, this study investigated the variability of compressive strength as a function of moisture content, recognizing that accurately defining this parameter in uncontrolled environmental conditions is both challenging and critical to ensuring the mechanical performance of CSRE.

**Table 1:** Methods of Specimen Preparation for CSRE Compressive Strength Testing.

Ref.	Sample Geometry	Compaction Method	Binder Type & Content	Moisture Content	Curing Conditions	Compressive Strength (MPa)	Soil Processing Description	Soil Condition
[15]	Cubes (100 × 100 × 100 mm)	Mechanical ramming	Cement (3–10%)	6–14%	28 days at ambient conditions	2.4–13.0	*Soil sieved and blended with sand and gravel to achieve desired granulometry.*	mechanically processed soil
[16]	Cubes and cored cylinders	Mechanical ramming	Cement (10–20%)	Optimum moisture content via Proctor test	120 days at ambient conditions	Varies; strength increases over time	*Soil stabilized with cement; specimens cured for extended periods to assess strength development.*	no information
[17]	Cylinder (Ø150 × 150 mm)	Manual compaction	None; straw fibers (0.1–0.4%)	Moisture content of the raw material 15.33%	28 days at ambient conditions	Up to 1.5	*Natural red soil mixed with straw fibers of varying lengths and contents.*	natural soil
[18]	Prisms (40 × 40 × 160 mm)	Mechanical ramming	None	9.58–9.9%	28 days at ambient conditions	Approximately 1.5	*Soil sieved and blended with sand to optimize particle size distribution.*	mechanically processed soil
[19]	Cubes (100 × 100 × 100 mm)	Mechanical ramming	Biopolymers (e.g., xanthan gum)	Not reported in source study	28 days at ambient conditions	1.2–2.0	*Natural soil stabilized with biopolymers to enhance cohesion and water resistance.*	natural soil
[4]	Cubes (100 × 100 × 100 mm)	Mechanical ramming	Cement (6%)	7–8%	28 days at ambient conditions	3.0–5.5	*Soil mineralogy varied; samples included montmorillonite, beidellite, and kaolinite.*	no information
[20]	Cylinder (Ø150 × 150 mm)	Mechanical compaction	None; reinforced with straw fibers	8.9%	28 days at ambient conditions	0.75–2.88	*Local soil mixed with straw fibers; focus on optimizing workability and mechanical properties.*	natural soil
[21]	Various (cubes, cylinders)	Manual and mechanical ramming	None and various stabilizers	0.7 to 12.0%	Varies (up to 90 days)	1.0–3.5	*Studies conducted on traditional and modern rammed earth structures with varying compositions.*	no information
[22]	Cylinder (Ø50 × 100 mm)	Mechanical compaction	None	Optimum water content	28 days at ambient conditions	Approximately 1.0	*Soil compacted at optimum moisture; samples cored from larger Proctor molds.*	no information
[23]	Not specified	Manual compaction with varying loads (1–3 MPa)	Polymer aqueous solution; cement	10% and 20%	1, 3, and 7 days at ambient conditions	1.7–2.4	*Red soil mixed with polymer aqueous solution and cement; moisture content adjusted to 10% or 20%.*	no information
[24]	Cube (50 × 50 × 50 mm)	Autonomous mechanical compaction	Epoxy emulsion (6.8%)	Not reported in source study	3, 6, 12, and 24 h at ambient conditions	Up to 2.4	*Red soil stabilized with epoxy emulsion to enhance early-age strength for automated construction.*	no information
[25]	Not specified	Mechanical compaction	Liquid polymer (various percentages)	Optimum moisture content via Proctor test	7 days-open air	Over 13	*Natural soil modified with liquid polymer; optimum moisture content determined for stabilization.*	natural soil
[26]	Cylinder (dimensions not specified)	Manual or pneumatic ramming	Natural mining by-products	6.9–21.4%	7.28 and 90 days ambient condition	0.6–12.5	*Soil mixtures incorporating mining by-products evaluated for suitability in rammed earth construction.*	no information
[27]	40 × 40 × 160 mm	Not reported in source study	Cement (2–8%)	18% (including the residual moisture content in the soil)	28 days	2.0 ± 0.2 (with 2% cement)	*Soil stabilized with varying cement contents; compressive strength assessed using rebound hammer test.*	no information
[28]	Cubes (70.7 mm, 100 mm, 150 mm) and cylinders	Not reported in source study	None	18.2–23%	14, 21, and 28 days at ambient conditions	Not reported in source study	*Soil passed through a 2-mm sieve to remove debris; uniaxial compression tests conducted to develop constitutive equations.*	no information
[29]	Not specified	Mechanical compaction	Natural stabilizers (e.g., agricultural by-products)	Not specified	Not specified	Not specified	*Soil mixed with local waste and recycled materials to enhance mechanical strength without compromising recyclability.*	no information
[30]	Blocks (10 cm (depth) × 20 cm (length) × 30 cm (height))	Manual compaction	None	Controlled moisture content 14.0%	Not reported in source study	0.9–4.17	*Soil compacted using historical techniques; study highlights variability in compressive strength across different wall regions.*	no information
[31]	Small cylinders (diameter 10.1cm, height 11.5cm) Prisms (50 × 50 × 10 cm)	Mechanical compaction	None	Optimum moisture content 13%	Not reported in source study	1.36–1.4	*Unstabilized soil compacted into prismatic samples; mechanical properties assessed through experimental and numerical methods.*	no information
[32]	Cubes (150 mm)	Mechanical compaction	Metakaolin (various percentages)	Determined via compaction tests	7, 14, and 28 days at ambient conditions	0.3–1.2	*Soil stabilized with metakaolin; compressive strength evaluated over different curing periods.*	no information
[33]	Not specified	Not specified	Various chemical stabilisers and fibres cement contents of 7% and 10% along with 0.5%, 1%, 1.5% and 2% fibre content	Not reported in source study	28 days	Highest value 6.87	*Comprehensive review of chemical stabilisation and fibre reinforcement methods to improve mechanical properties of rammed earth.*	no information
[34]	Cylinder for UCS (39.1 (diameter × 80 height, mm)	static compaction method per ASTM D 2166	Varied clay contents	optimum moisture Content via Proctor test	Ambient conditions in the laboratory 28 days	0.73–1.35	*Study on how varying soil content affects the engineering properties of unstabilized rammed earth.*	no information

The reviewed studies demonstrated that specimen geometry, slenderness ratio, binder percentage and compaction energy substantially influenced UCS results. Variations in these parameters contributed to the wide methodological diversity documented in the literature, limiting direct comparability between studies. The studies listed in Table 1 showed that the wide range of reported UCS values directly resulted from significant methodological differences rather than solely from soil properties. Variations in specimen geometry (cubes, prisms, cylinders) and slenderness ratios affected both end-confinement conditions and failure mechanisms. Compaction procedures also differed markedly: some studies used manual tamping, others applied mechanical ramming or pneumatic systems, resulting in different compaction energies and densities. Binder type and dosage ranged from unstabilized soils to mixtures containing cement, biopolymers or epoxy emulsions, each producing distinct strength development mechanisms. Moisture content—although critical to UCS—was not always reported, which limited comparability. Soil preparation techniques also varied, from minimally processed ‘as-dug’ soils to mixtures that were sieved, blended or milled, altering particle packing and internal structure. These methodological inconsistencies explained the broad variability in UCS reported across the literature and highlighted the need for standardized reporting to enable meaningful comparison between studies. This variability further justified the controlled approach adopted in this study, which isolated the effects of soil preparation, moisture content and cement dosage.

## 2. Materials and Methods

### 2.1. Materials

#### 2.1.1. Soils Used in the Study

**Figure 4:**
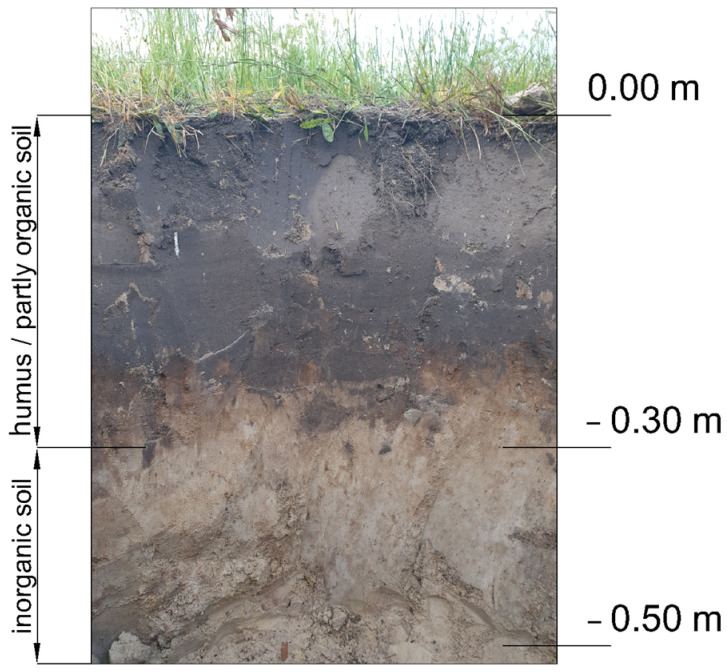
Photograph showing the geological cross-section of the soil profile. Native soil for laboratory tests was sampled from a depth of 50 cm below the ground surface—20 cm beneath the humus and partly organic soil layer for rammed earth production. The soil was sampled from a depth of 50 cm below the ground surface, excluding the 20 cm thick humus layer.

The soil for the study was collected in Inwałd (Lesser Poland Voivodeship, southern Poland). According to the requirements of NZS 4298:1998 [11], soils containing organic matter are not permitted; therefore, samples were taken from a depth of 0.5 m below ground level, i.e., approximately 20 cm beneath the humus layer (Figure 4). Moisture content was determined gravimetrically by drying the material at 105 °C to constant mass and calculating the water fraction relative to the dry mass, in accordance with standard laboratory soil-testing practice.

In the experimental program, the same natural soil was used, prepared in two different variants to assess the influence of material preparation on the properties of cement-stabilized rammed earth (CSRE) mixtures.

The first variant of material consisted of soil in its natural moisture state, collected directly from the site. In this study, the term natural soil refers to the native material used in its moist state, with natural soil preserved. Its initial moisture content was determined by drying a representative sample at 105 °C to constant mass. The moist bulk material was stored in a tightly sealed container to maintain its moisture level until mixture preparation. This variant reflects field-oriented practice, where soil is applied in aggregated form without extensive mechanical processing.

**Figure 5:**
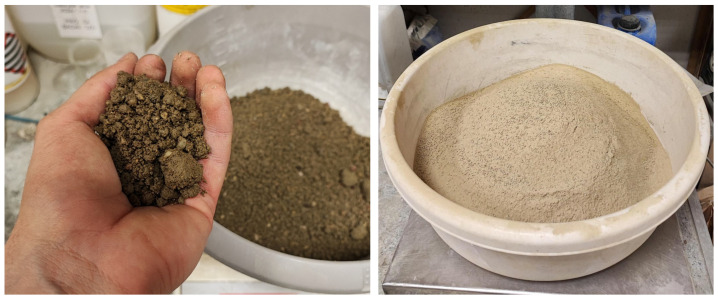
**Left**: natural soil; **Right**: powdered soil obtained through wet separation and manual grinding of the fine fraction in a mortar, with reconstitution of the original grain-size distribution.

The second variant, hereafter referred to as powdered soil, was prepared by initially separating the coarse fraction using the wet method in accordance with ISO 17892-4:2016 [35], which involves washing the sample on a 0.063 mm sieve. The resulting suspension was evaporated and subsequently dried to a constant mass, after which the material was manually powdered in a mortar. The fine fraction obtained in this way was then combined with the previously separated coarse fraction (retained on the sieve) and thoroughly mixed to reconstitute the original grain-size distribution of the soil. This procedure was used to prepare the entire batch of powdered soil employed for specimen preparation in this study. Both preparation variants of the soil material are presented in Figure 5.

**Figure 6:**
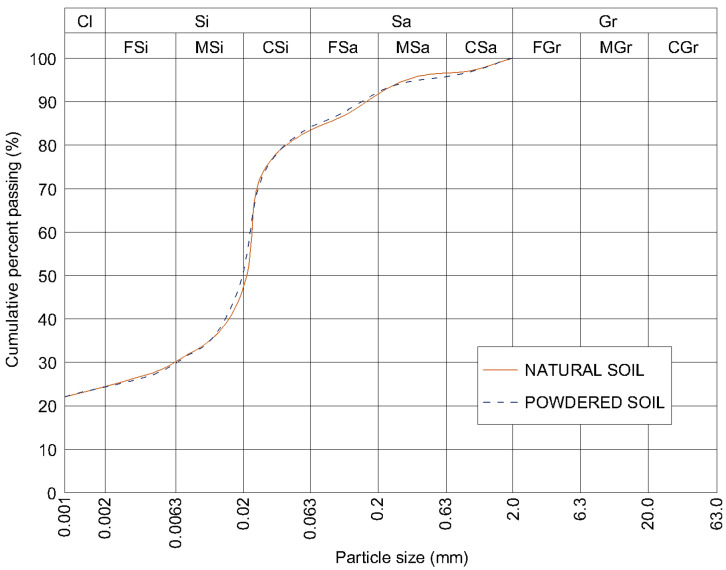
Grain size distribution of the tested soil: natural soil versus powdered soil.

To verify the homogeneity of the prepared material and to assess whether the powdered soil differed granulometrically from the natural soil, three subsamples were collected from the thoroughly mixed natural soil and three from the thoroughly mixed powdered soil. For each series, three hydrometer analyses were performed in accordance with ISO 14688:2017 [36]. The average results of these analyses are presented in Figure 6. The findings confirmed that the applied powdering process did not alter the original grain-size distribution of the soil.

Mineralogical composition is a primary determinant of compressive strength development in cement-stabilized rammed earth (CSRE), as documented in the literature [4]. To ensure comparability between the results obtained in this study and those reported by other authors, the mineralogical composition of the native soil was determined using thermal analysis.

**Figure 7:**
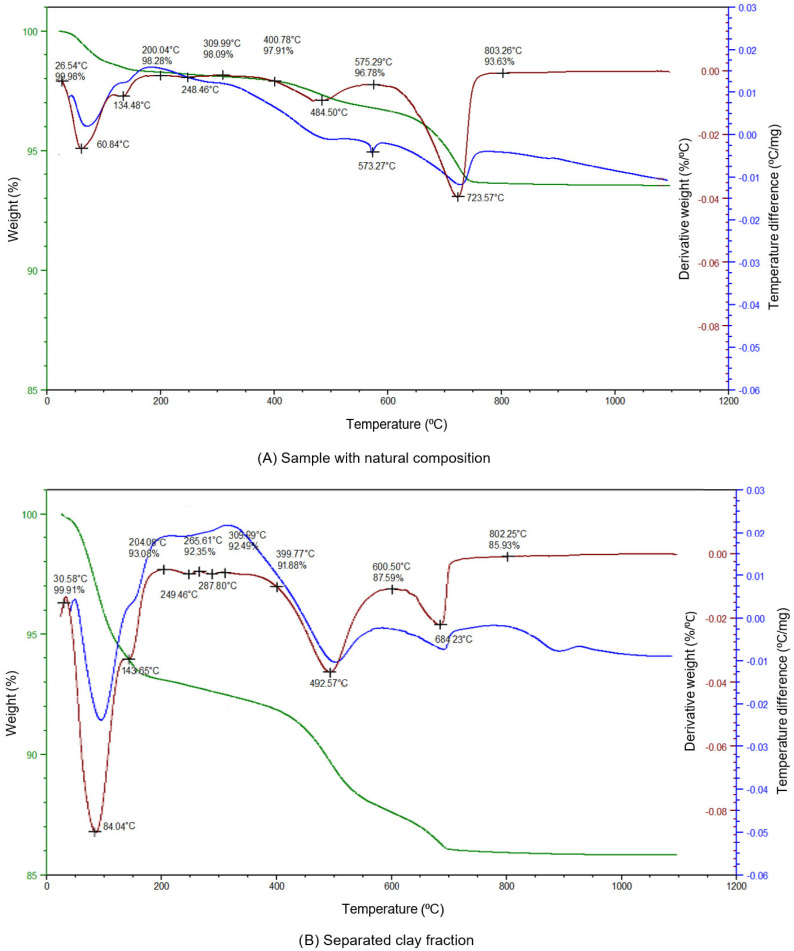
Thermogravimetric–differential thermal analysis results for the investigated material. The left plot (**A**) corresponds to the raw soil sample with its natural composition, while the right plot (**B**) shows the behaviour of the separated clay fraction under identical thermal conditions.

Thermal analysis was performed with a Q600 analyser (TA Instruments, New Castle, DE, USA). The following test conditions were applied: sample mass 80–100 mg, instrument-controlled automatic sensitivity, heating rate 10 °C/min, atmospheric environment—air. The resulting thermograms for the raw sample (a) and the separated fraction (b) (Figure 7) exhibit characteristic thermal events, including dehydration of clay minerals in the range 25–200 °C, goethite dehydration at approximately 200–300 °C, dehydroxylation of clay minerals between 400 and 580 °C, and thermal decomposition of calcite within 580–800 °C.

The analysed natural soil is characterized by a high content of clay minerals (24.3%, Table 2). Quartz is the predominant phase (66.9%), while illite constitutes the dominant clay mineral (17.4%). Additional clay minerals include beidellite (4.7%) and kaolinite (2.2%). Non-clay minerals identified in the sample comprise calcite (7.0%) and goethite (1.8%).

The thermogram exhibits multiple characteristic thermal events for the raw soil sample. An initial mass loss between approximately 25 and 200 °C is associated with the dehydration of adsorbed and interlayer water in clay minerals. A subsequent endothermic effect around 200–300 °C corresponds to the dehydration of goethite. The broad dehydroxylation peak observed between 400 and 580 °C reflects the structural breakdown of clay minerals such as illite and kaolinite. A pronounced mass loss in the range 580–800 °C indicates the thermal decomposition of calcite, confirmed by the accompanying endothermic peak. The residual signal stabilises above 800 °C, consistent with completion of carbonate decomposition. The thermogram of the isolated clay fraction shows intensified and more sharply defined thermal events attributable to clay minerals. Dehydration in the 25–200 °C range is more pronounced due to the higher proportion of hydrophilic phases. The endothermic effect near 200–300 °C again reflects goethite dehydration, although with reduced amplitude relative to the raw soil. A dominant dehydroxylation peak appears between 400 and 600 °C, representing the breakdown of illite and kaolinite structures. Only a minor mass-loss event near 600–800 °C is visible, consistent with the significantly lower carbonate content of the separated fraction. After 800 °C the curves show limited further change, as most thermally active phases have decomposed.

#### 2.1.2. Rammed Earth Mix Design

The granulometric composition of soils is one of the most critical parameters determining the suitability of mixtures for rammed earth and cement-stabilized rammed earth (CSRE) applications. The proportions of clay, sand, and gravel fractions control not only the mechanical performance of the hardened material but also its durability under environmental stressors such as freeze–thaw cycles. Excessive clay content increases capillarity and susceptibility to frost damage, whereas adequate proportions of sand and gravel enable proper compaction, reduce shrinkage, and provide a stable load-bearing skeleton.

Numerous standards and guidelines emphasize the necessity of limiting the clay fraction. RILEM TC 274-TCE [37] and NZS 4298:1998 [11] recommend that the clay content should not exceed 15%, with an optimal range of 5–12% for cement-stabilized soils. Houben and Guillaud [38] argue that values above 12% lead to excessive capillarity and frost susceptibility, while contents closer to 5% enhance cement hydration efficiency and reduce water absorption, without compromising the minimal cohesion required for compaction.

Sand and gravel fractions are equally crucial for strength and dimensional stability. According to NZS 4298:1998, the total proportion of gravel (>20 mm) should be limited to 30%, with 20–40% recommended for the 2–20 mm fraction and 25–75% for sand (0.06–2.0 mm) [11]. RILEM TC 274-TCE [37] suggests 30–40% gravel, 40–60% sand, and 10–20% fines, while Houben and Guillaud [38] propose up to 40% gravel, 25–80% sand, and 10–30% silt and clay. ASTM D1633 [39] guidelines emphasize the need for well-graded soils to achieve high density, a stable skeleton, and resistance to freeze–thaw cycling.

**Figure 8:**
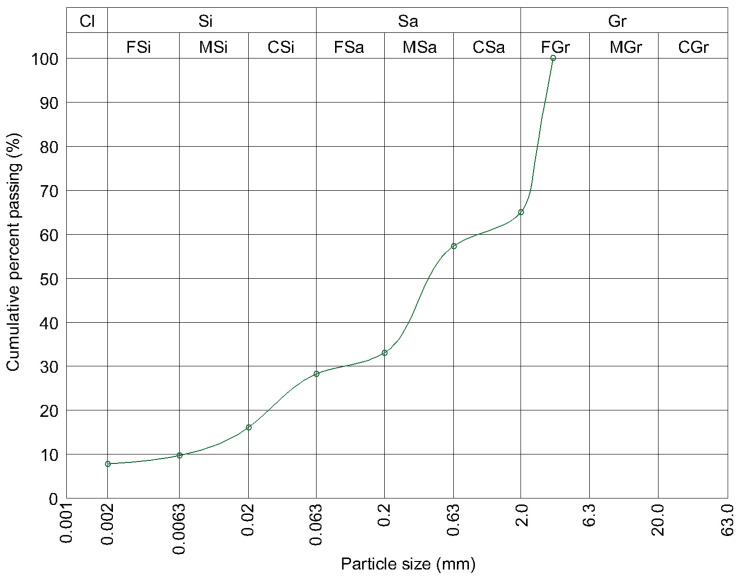
Grain size distribution of the natural soil after supplementation with controlled sand and gravel fractions.

To achieve the required grain-size distribution, dried sand and gravel were added to both the natural and the powdered soil. Since the detailed granulometric analysis revealed no significant differences between the natural and powdered soil, identical amounts of sand and gravel were incorporated into each material. Figure 8 presents the grain-size curve of the modified natural soil. The differences between the curves of the two modified soil mixtures were minimal, and the purpose of the figure is to illustrate the general characteristics of the soil mixtures used in the study.

In terms of mineral composition, the sand consisted of pure quartz. The gravel fractions contained 75% quartz and 25% carbonate clasts by mass. The mineral composition of the entire modified soil is presented in Table 3.

For the compressive strength evaluation, six series of cement-stabilized rammed earth (CSRE) specimens were prepared, differing in soil preparation method, cement content, curing time, and loading direction (Table 4). Two soil preparation methods were applied: powdered soil and natural soil. Cement contents of 7%, 9%, and 12% were used at a moisture content of 9%, 11% and 13%.

Curing time was set to 28 days for the majority of specimens, which corresponds to the standard reference period for assessing cementitious materials, while series 5a was tested after only 1 day to evaluate early-age strength development.

Six series were selected to isolate the independent effects of moisture, cement content and soil preparation while maintaining a feasible total specimen count. The loading direction during compressive strength testing was either perpendicular (“1”) or parallel (“0”) to the layering direction formed during compaction, allowing for the assessment of potential mechanical anisotropy of the material. Sample IDs followed the format: [Series]-[soil type]-C[cement content %]-W[moisture %].

### 2.2. Methods

#### 2.2.1. Specimen Preparation

The specimen preparation procedure for laboratory testing was designed to closely replicate the real construction conditions of vertical rammed earth elements. To ensure a compaction method representative of field practice, a manual rammer was developed, suitable for both laboratory use and full-scale construction of load-bearing rammed earth walls. The rammer had a mass of 6.5 kg and a compacting face of 96 × 96 mm.

**Figure 9:**
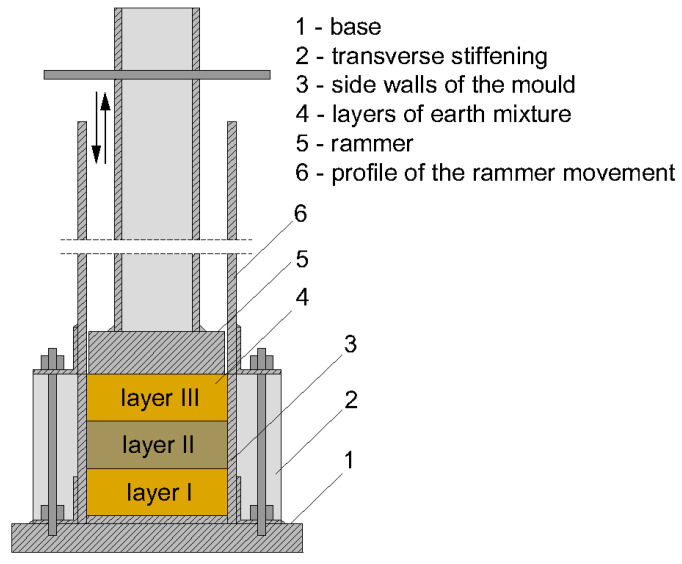
Schematic representation of the compaction procedure used for the preparation of test specimens. Mold internal dimensions: 100 × 100 × 100 mm. Guide collar height: 400 mm.

**Figure 10:**
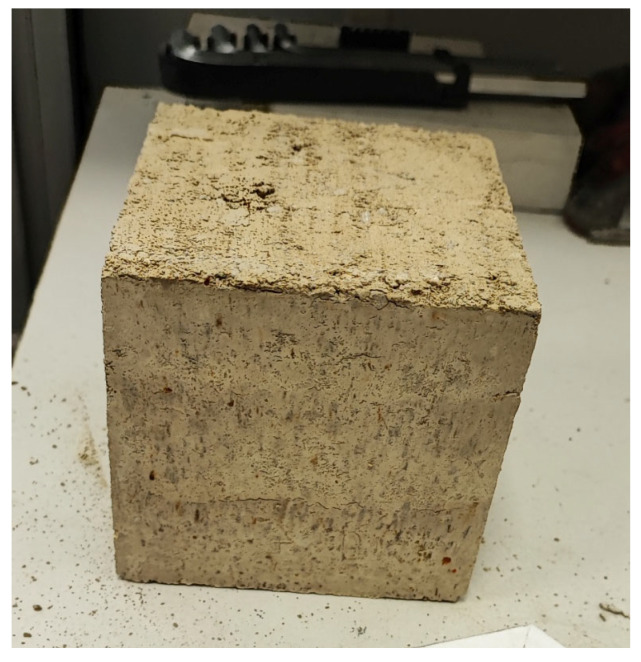
Cement-stabilized rammed earth specimen showing three distinct compacted layers.

The specimens were prepared in steel cubic molds with internal dimensions of 100 × 100 × 100 mm. The soil mixture was compacted by repeatedly dropping the rammer freely from a height of 30 cm onto the surface of the moist earth mixture. To ensure a vertical drop path, a steel guiding collar was attached to the top edge of the mold. For mechanical testing purposes, the specimens were formed in three equal layers (Figure 9). Each layer was compacted by twenty consecutive drops of the rammer from a height of 30 cm. This corresponded to a compaction energy of 38.2 J per blow (6.5 kg × 0.30 m) applied 20 times per layer. This procedure is consistent with current laboratory practices for the preparation of rammed earth specimens [40,41]. An exemplary CSRE specimen prepared by layered compaction is presented in Figure 10.

The specimens were demolded after 24 h and subsequently cured under controlled conditions at a relative humidity of 75% and a temperature of 20 °C. A lower relative humidity than that typically used for concrete specimen curing was intentionally selected, as it more accurately reflects the hygrothermal conditions commonly encountered on construction sites involving earthen materials [42,43]. This approach allows for a more representative assessment of the degree of cement hydration in CSRE specimens under practical curing environments.

#### 2.2.2. Optimum Moisture Content of the CSRE Mixture

The moisture contents selected for specimen preparation were positioned around the optimum determined by the modified Proctor test to examine the sensitivity of UCS to deviations from peak compaction conditions. The moisture content of CSRE mixtures is a key parameter influencing the mechanical performance of the hardened material. Hall and Djerbib [44] demonstrated that molding specimens at the optimum moisture content allows for the highest bulk density to be achieved, which directly translates into greater compressive strength. Conversely, Beckett and Ciancio [45] observed that maximum strength values may occur at moisture contents slightly below the optimum.

**Figure 11:**
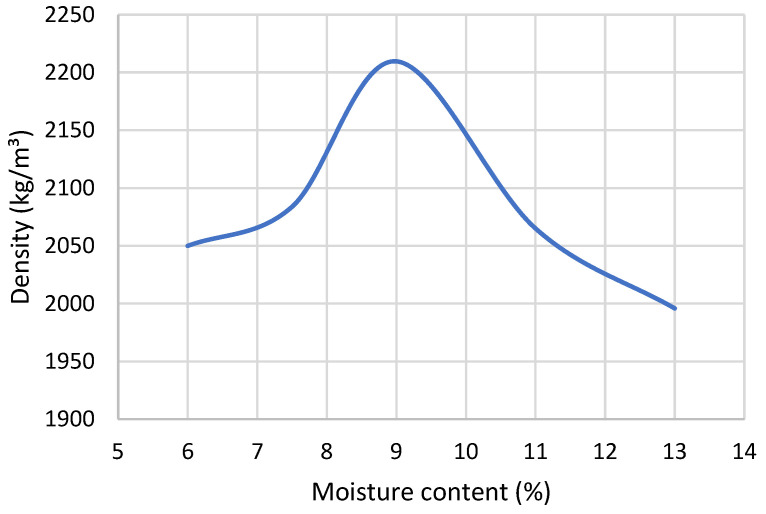
Compaction curve used to determine the optimum moisture content of CSRE mixture (modified Proctor test).

In the present study, a modified Proctor test was conducted using a 6.5 kg hand rammer, typically employed for CSRE specimen preparation, to determine the optimum moisture content of the soil mixture composed of natural soil stabilized with 9% CEM I 42.5R cement (Figure 11). The modified Proctor method was used because it employed the same rammer and compaction mechanism as the specimen preparation process.

#### 2.2.3. Ball Drop Test for CSRE

**Figure 12:**
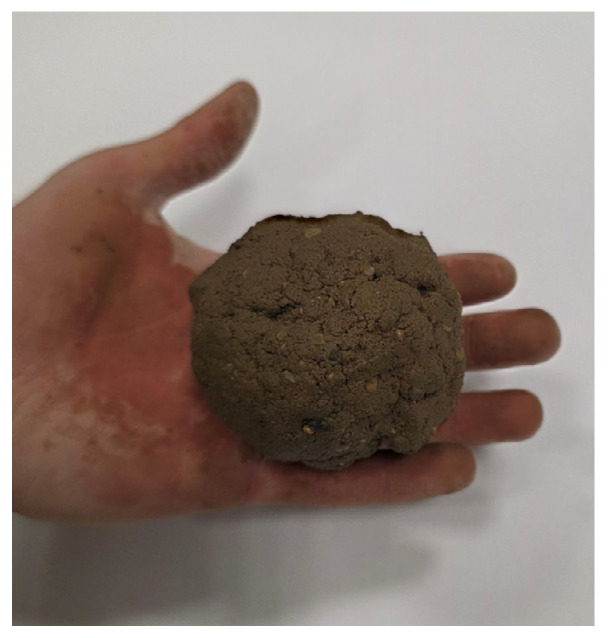
Ball prepared for the drop-ball test in accordance with NZS 4298:1998 [11].

For comparison, a drop-ball test was additionally performed on CSRE mixtures stabilized with 9% cement and prepared at varying moisture contents (Figure 12). This approach enabled a direct evaluation of the correspondence between the empirical field-based method and the standardized laboratory compaction procedure. The results demonstrated consistency with the requirements of NZS 4298:1998 [11], as the ball prepared at the optimum moisture content disintegrated in a manner most closely matching the normative recommendation (Table 5). These findings confirm the reliability of the drop-ball test as an empirical method for identifying the optimum water content of CSRE mixtures.

#### 2.2.4. Compressive Strength Test

Compressive strength tests on CSRE cube specimens (100 × 100 × 100 mm) were conducted using a testing machine (Controls Automax, Milan, Italy) with a 0–3000 kN capacity, following the general principles of EN 12390-3:2019 [46], adapted for earthen materials. Due to the layered structure of CSRE, specimens were loaded in the direction of compaction, except for one series in which the load was applied parallel to the layering. The specimens predominantly failed in a brittle-shear mode, forming a characteristic “double frustum” shape, consistent with literature on cement-stabilized earthen materials. Cube specimens exhibited a slenderness ratio of 1.0.

#### 2.2.5. Theoretical Background on Statistical Methods Used

This study employed a suite of parametric statistical techniques to examine the influence of processing variables on unconfined compressive strength (UCS), while ensuring adherence to the underlying assumptions required for valid inference. In the context of materials testing, especially when working with small sample sizes, any internal variation unrelated to the variable under investigation can mask real effects or introduce misleading signals. Therefore, for each statistical comparison performed in this study, groups were defined based on controlled experimental conditions such as identical moisture content, cement dosage, curing time, or load application method. This approach ensured that any observed differences in unconfined compressive strength (UCS) could be more confidently attributed to the targeted variable, rather than to uncontrolled or interacting factors.

To assess the normality of UCS distributions within predefined groups, the Shapiro–Wilk test was utilized [47]. It is considered one of the most powerful normality tests for small to moderate sample sizes and is widely used in engineering statistics to validate assumptions for parametric analysis [48].

Verification of variance homogeneity between groups was conducted using Levene’s test, which evaluates whether variances are equal across samples [49]. Homogeneity of variances is a critical assumption for standard ANOVA procedures. If this assumption is violated (i.e., *p* < 0.05), the use of classical ANOVA may lead to incorrect conclusions, requiring more robust alternatives.

For group comparisons where assumptions were met, a one-way Analysis of Variance (ANOVA) was applied, enabling the detection of significant differences in mean response values (here, UCS) across groups formed by categorical factors [50]. In cases where Levene’s test indicated variance heterogeneity, the analysis employed Welch’s ANOVA or the Welch *t*-test [51], which are designed to correct for inequality of variances while maintaining statistical power [52].

Finally, Pearson’s correlation coefficient was used to quantify the strength and direction of linear relationships between continuous variable, bulk density, and UCS [53]. This analysis supported the identification of variables that, while not manipulated directly, exhibited strong association with mechanical behaviour [54].

The dataset analyzed in this study consists of 30 samples, each characterized by a set of geotechnical and processing variables along with corresponding unconfined compressive strength (UCS) values. The data are organized in tabular form and include measurements of soil type, cement content, moisture content, curing time, direction of load application and bulk density. UCS serves as the response variable.

To preserve the validity of parametric tests such as ANOVA and correlation analysis, it was occasionally necessary to exclude specific samples from the dataset—particularly those with atypical characteristics that would confound results. For example, samples cured for only 1 day consistently exhibited mechanical behavior unrepresentative of the general trends, likely due to incomplete hydration processes. Their inclusion would have reduced statistical power, and obscured real relationships. These decisions were made transparently and systematically, as reflected in the group definitions accompanying each analysis.

In this study, the goal of the statistical analysis was not to establish certified characteristic values of material parameters in accordance with technical standards, but rather to identify trends and assess the significance of relationships between processing variables and unconfined compressive strength (UCS). For this purpose, small group sizes—even only three to five samples per group—are methodologically acceptable, provided that the data are internally consistent and the experimental design is well-controlled.

Normative protocols typically require larger sample sizes to determine characteristic values (e.g., 5th percentile strength) or to validate conformity with specification thresholds. In contrast, the present analysis aims to understand which factors affect UCS and how, rather than to certify the material for use. A significance level of 0.05 (corresponding to a 95% confidence level) was adopted for all statistical analyses. This threshold is widely recommended in experimental research to balance the risk of Type I errors while maintaining sufficient power for detecting true effects [55,56]. These methods were selected because they remained statistically appropriate for the limited specimen count inherent to CSRE preparation and curing.

## 3. Results

A total of 30 unconfined compressive strength (UCS) tests were performed on cement-stabilized rammed earth specimens, each prepared according to controlled variations in material composition and curing conditions. All specimens were subjected to the same loading procedure under standardized conditions to ensure consistency in test execution and result comparability. Table 6 presents the full dataset, including key input variables—such as cement content, moisture content, curing time, soil form, and load direction—along with the corresponding UCS values obtained from each test. This comprehensive matrix serves as the empirical foundation for the subsequent statistical evaluation of influencing factors. The wide strength range reflected combined effects of curing duration, moisture variation and cement content.

Despite the relatively small sample size, the design ensures a representative distribution across several key processing parameters, including cement content (ranging from 7% to 12%), moisture content (9% to 13%), and curing time (1 to 28 days). Soil type is binary (0—natural soil, 1—soil powder), with powdered soil representing 20% of the dataset. Most samples (90%) were tested under perpendicular loading, while a minority (10%) were loaded parallel to the specimen axis. This experimental variety allows for evaluating both material composition and procedural influences on compressive strength.

The unconfined compressive strength (UCS) across all samples ranges from 3.62 MPa to 29.06 MPa, with a mean value of 13.42 MPa and a standard deviation of 7.04 MPa. This wide spread indicates substantial variability in mechanical performance, directly reflecting differences in preparation parameters. Notably, the lower quartile (Q1 = 9.35 MPa) and upper quartile (Q3 = 15.46 MPa) indicate that 50% of the samples lie within a relatively moderate UCS range, whereas the extreme values highlight the strong impact of curing and binder content. Pre-test mass and density values display limited variation, with average values of approximately 2225 g and 2171.5 kg/m^3^, respectively. Standard deviations are relatively small (mass: ±74.3 g; density: ±93.6 kg/m^3^), suggesting uniformity in specimen geometry and preparation, supporting the validity of comparisons across other variables such as cement ratio and curing time.

Although the UCS range obtained in this study (3.62–29.06 MPa) appeared wide, such variability was consistent with previous research on cement-stabilized rammed earth. Earlier studies reported similarly broad strength intervals depending on cement dosage, compaction energy, moisture conditions and curing time. For example, UCS values in the literature range from below 2 MPa for unstabilized or early-age mixtures to well above 20 MPa for specimens with higher cement contents and extended curing. The present experimental program intentionally incorporated multiple cement levels, moisture contents and curing durations, including 1-day specimens, which further contributed to the expanded strength interval. Therefore, the observed UCS range aligned with established findings and reflected controlled variations in key processing parameters rather than experimental inconsistency.

Preliminary subgroup comparisons show that higher UCS values are consistently associated with 12% cement content, moisture levels maintained at 9%, and a curing time of 28 days. In contrast, the lowest strengths are observed in samples cured for only 1 day, irrespective of other variables. Furthermore, samples with force applied perpendicularly tend to produce more reliable and higher UCS readings, reinforcing the significance of standardized loading procedures. These group-level trends establish a strong foundation for the inferential statistics applied in the subsequent sections of the study.

## 4. Results Analysis

### 4.1. Effect of Soil Type

The comparison between powdered soil and natural soil (with natural clods preserved) addressed the central methodological question of this study. To ensure validity, only specimens with identical cement content and moisture level were considered, thereby isolating the effect of soil preparation method.

The Shapiro–Wilk test confirmed normality of UCS distributions across groups (*p* > 0.05), and Levene’s test supported variance homogeneity (*p* > 0.05). The ANOVA results further indicated no statistically significant differences in UCS between the two soil preparation variants (*p* > 0.05).

**Figure 13:**
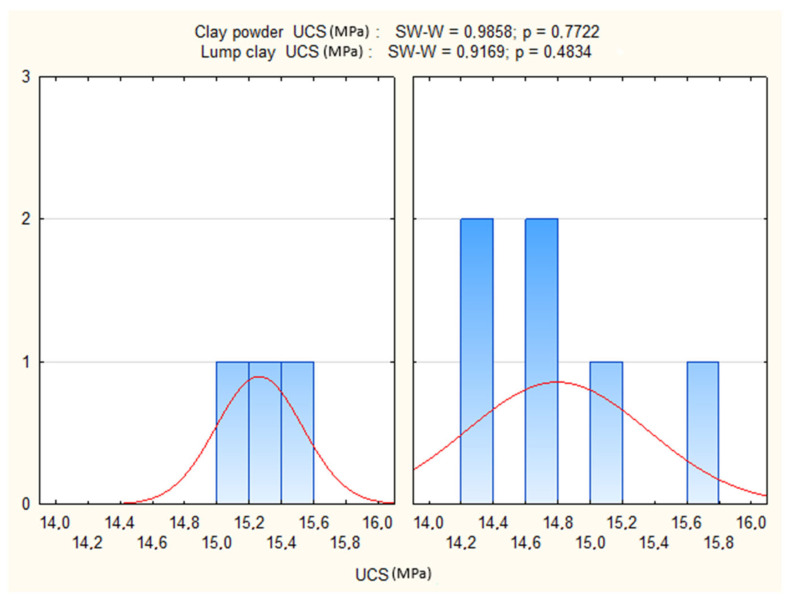
Distribution of UCS values and results of the Shapiro–Wilk normality test for natural and powdered soil specimens.

**Figure 14:**
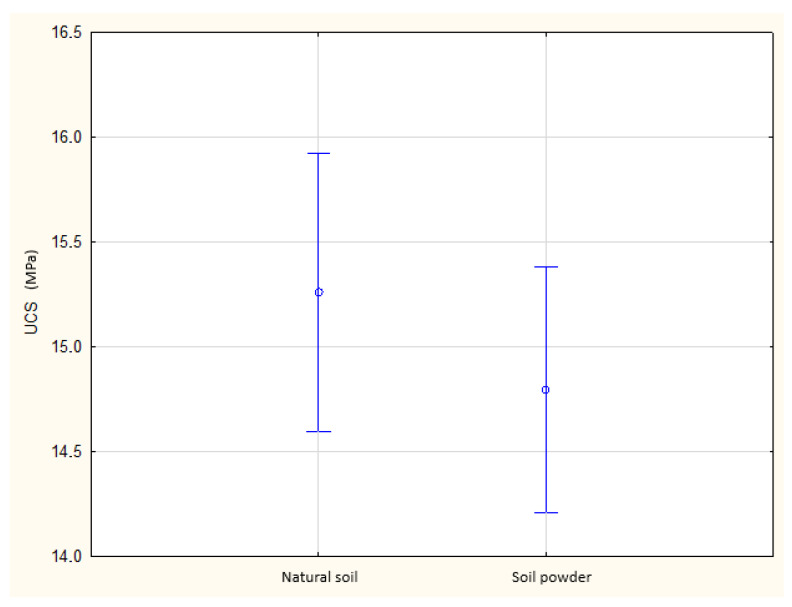
Comparison of mean UCS values with 95% confidence intervals for natural and powdered soil specimens.

**Figure 15:**
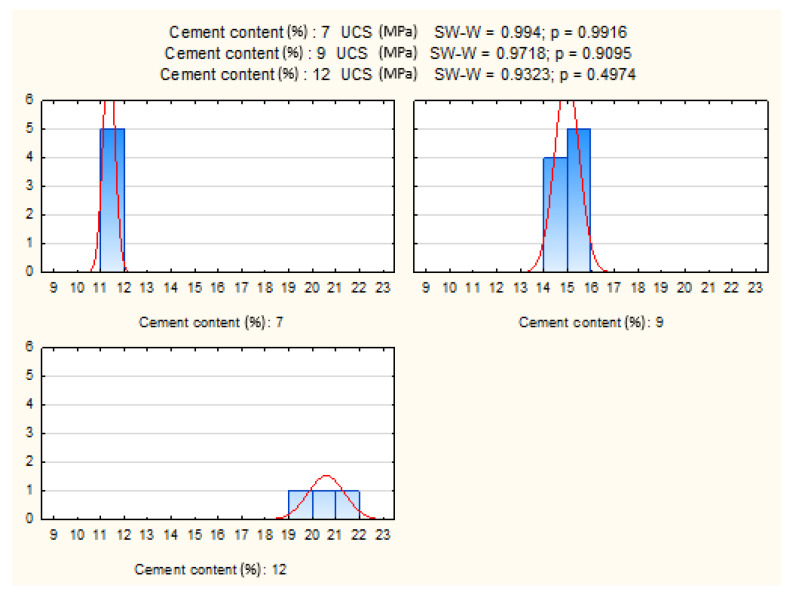
Effect of soil type on UCS: absence of statistically significant differences under constant cement and moisture conditions.

Figure 13 presents the UCS distributions and the outcomes of the Shapiro–Wilk test for both soil types, showing that statistical assumptions were satisfied. Figure 14 compares the mean UCS values with 95% confidence intervals, clearly demonstrating the absence of significant differences between powdered and natural soil. Figure 15 graphically illustrates this null effect, reinforcing the conclusion that soil powdering does not influence UCS under controlled cement and moisture conditions.

This null result represents the key outcome of the research. It demonstrates that soil powdering—commonly applied in laboratory studies—is not a determining factor for compressive strength. The granulometric composition remains unchanged, and the removal of natural aggregates does not produce measurable strength benefits. Instead, moisture content, cement dosage, and curing time are confirmed as the dominant variables.

From a practical perspective, avoiding powdering reduces specimen preparation time and costs, while aligning laboratory procedures more closely with real construction practice. This highlights the importance of focusing on precise moisture control rather than on labor-intensive soil pre-processing when evaluating or applying cement-stabilized rammed earth. Although the number of specimens in each soil-type subset was limited, statistical evaluation was feasible because the compared groups shared identical moisture content, cement content, curing time and loading direction. These controlled conditions ensured internally consistent datasets, and both the Shapiro–Wilk and Levene’s tests confirmed that the assumptions required for parametric analysis were satisfied. Consequently, a one-way ANOVA could be applied, and the analysis showed no statistically significant effect of soil preparation method on UCS. Therefore, the statistical assessment of soil type was not hindered by sample size or data structure.

### 4.2. Effect of Cement Content

The influence of cement dosage was examined by selecting specimens with identical moisture content, curing duration, and load application direction. Normality and homogeneity of variance were confirmed (*p* > 0.05).

**Figure 16:**
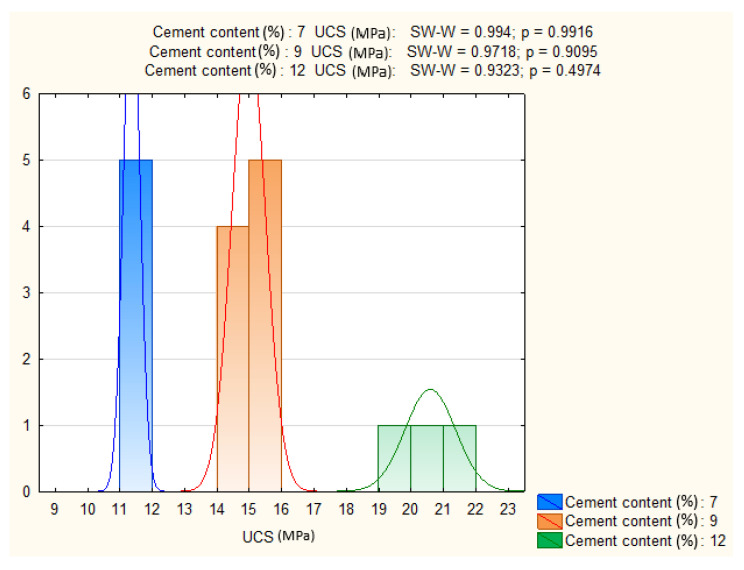
Shapiro–Wilk test results and UCS distributions for three cement contents.

**Figure 17:**
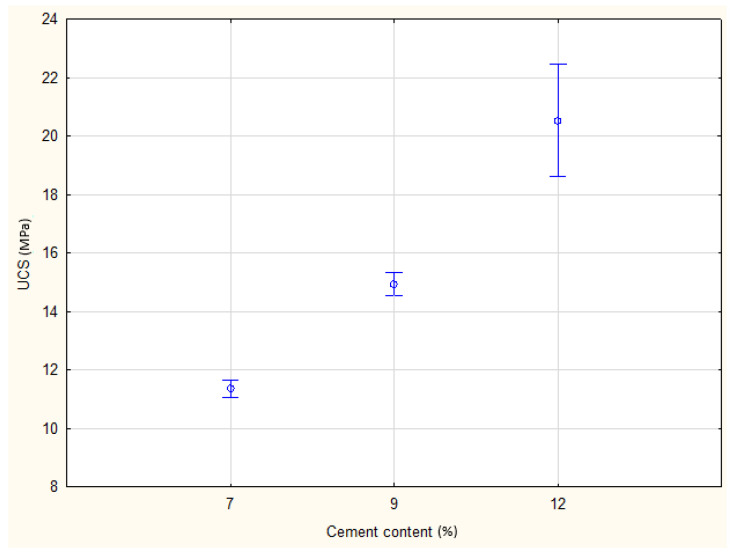
Comparison of mean UCS values with 95% confidence intervals for different cement contents.

ANOVA revealed a highly significant effect of cement content on UCS (*p* = 0.000), confirming that strength development is strongly dependent on binder proportion. Figure 16 presents the distributions, while Figure 17 shows mean UCS values with confidence intervals. The results indicate a systematic increase in UCS with higher cement dosages, consistent with expected mechanical improvements from cement stabilization.

### 4.3. Effect of Moisture Content

To investigate moisture effects, the dataset was filtered to include only specimens containing 9% cement, cured for 28 days, and tested under standard load orientation. This ensured that observed differences could be attributed solely to moisture variation.

**Figure 18:**
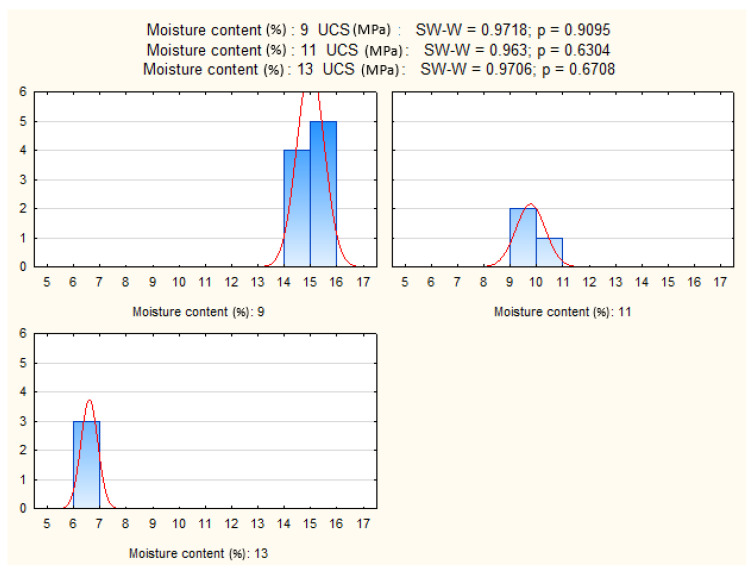
UCS distributions of CSRE specimens prepared with different moisture contents.

**Figure 19:**
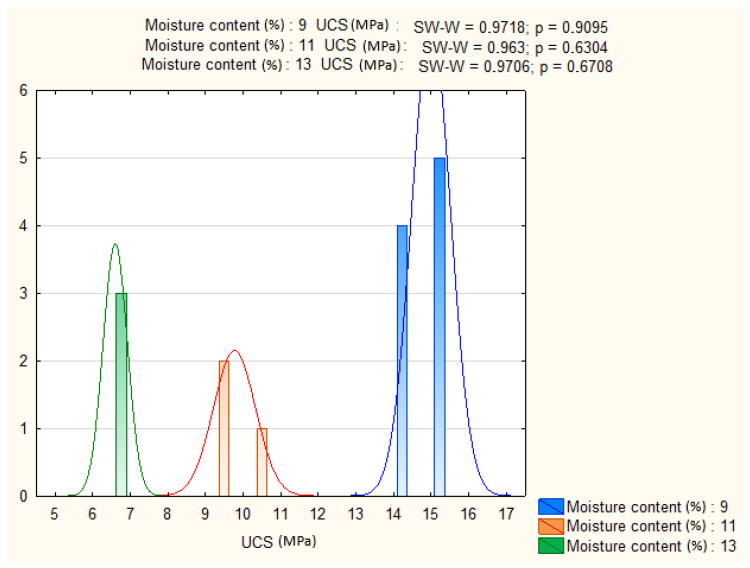
Shapiro–Wilk test results and UCS histograms for three moisture content levels.

The Shapiro–Wilk and Levene’s tests confirmed normality and homogeneity of variances (*p* > 0.05), satisfying ANOVA assumptions (Figure 18 and Figure 19). The analysis revealed a highly significant effect of moisture content on UCS (*p* ≈ 0). This result confirms the strong sensitivity of UCS to moisture conditions during sample preparation, underscoring the importance of precise water control in achieving target mechanical performance.

The results obtained from the modified Proctor test and the drop-ball test were consistent. The modified Proctor procedure identified the moisture level at which the maximum dry density was achieved, and specimens compacted at or slightly below this optimum exhibited the highest UCS values. The drop-ball observations qualitatively reflected the same trend: mixtures prepared at the Proctor-determined optimum moisture fragmented into several large pieces, as required by NZS 4298:1998, while mixtures wetter or drier than the optimum either remained intact or shattered into excessively fine particles. These findings indicated that the modified Proctor test provided the quantitative benchmark for optimum moisture content, whereas the drop-ball test served as a qualitative field-verification method rather than a substitute for laboratory calibration.

### 4.4. Effect of Curing Time

The effect of curing duration was assessed by comparing specimens with 12% cement cured for 1 day and 28 days. While the Shapiro–Wilk test confirmed normality (*p* > 0.05), Levene’s test indicated a violation of homogeneity (*p* ≈ 0). Therefore, Welch’s ANOVA and Welch’s *t*-test were applied.

**Figure 20:**
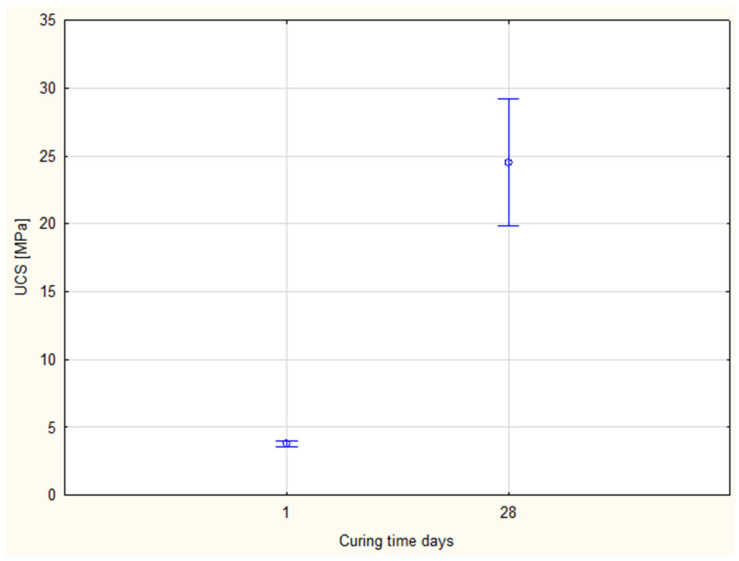
Comparison of mean UCS values with 95% confidence intervals for different curing times.

Both methods confirmed a highly significant effect of curing time on UCS (Welch’s ANOVA: F = 132.04, *p* = 0.00085; standard ANOVA: F = 84.60, *p* = 0.000016). The large F-values highlight the strong influence of curing duration on compressive strength. Confidence intervals (Figure 20) reinforce the reliability of Welch’s correction in cases of unequal variance.

### 4.5. Correlation Analysis of Physical Parameters

Pearson’s correlation was applied to assess the relationship between pre-test density, and UCS. Across the full dataset, both parameters showed weak linear associations with UCS (r = 0.200). However, after excluding specimens cured for only 1 day, correlations improved markedly (r = 0.758). This change indicates that immature specimens introduced disproportionate variability, likely due to incomplete cement hydration. Samples cured for 1 day (series 5a: specimens 5a-w-C12-W9-1 to 5a-w-C12-W9-4) were excluded from correlation analysis due to immature hydration, which introduced non-representative variability.

Further refinement of the dataset—by removing 28-day samples tested under parallel loading—yielded r = 0.853 These results highlight that pre-test density is a strong predictor of UCS and most probably reflects compaction quality.

**Figure 21:**
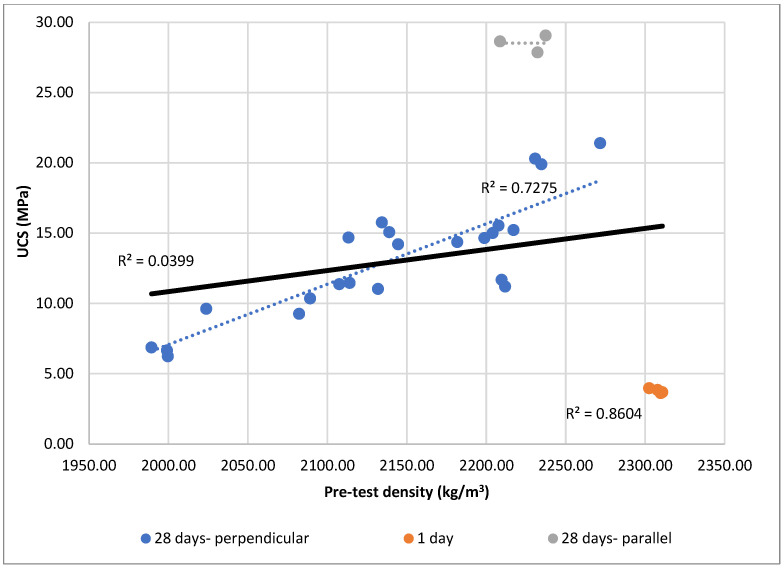
Relationship between pre-test density and UCS for CSRE specimens.

Complementary trendline analysis confirmed these patterns. For pre-test density (Figure 21), the global R^2^ was low (0.0399), but within-group relationships were much stronger: 28-day perpendicular (R^2^ = 0.7275) and 1-day specimens (R^2^ = 0.8604). By contrast, the 28-day parallel group showed no correlation (R^2^ ≈ 0), likely due to low variability in both UCS and density.

Overall, both correlation and regression analyses consistently indicated that density provides a reliable indicator of UCS. This outcome aligns with physical reasoning, as density captures compaction and material uniformity.

### 4.6. Effect of Loading Direction

The influence of loading direction during compressive testing was assessed using specimens cured for 28 days and stabilized with 12% cement. This group, representing fully matured samples, was selected to evaluate this procedural factor. The Shapiro–Wilk test confirmed the normality of UCS distributions across loading directions (*p* > 0.05), while Levene’s test verified homogeneity of variances (*p* > 0.05), thereby fulfilling ANOVA assumptions. The subsequent analysis of variance revealed a statistically significant effect of loading orientation on UCS (*p* < 0.05). These findings demonstrate that loading configuration exerts a measurable influence on compressive strength values, underscoring the necessity of standardized testing procedures in the assessment of cement-stabilized soils.

## 5. Discussion

Table 7 summarizes the methodological approach and statistical outcomes and presents a structured overview of all variables examined in relation to unconfined compressive strength (UCS). For each factor, the corresponding subset of samples, the outcome of assumption checks (Shapiro–Wilk and Levene’s tests), and the results of ANOVA or Welch’s test (where applicable) are listed. Additionally, variable with measurable linear associations to UCS are accompanied by Pearson correlation coefficients, shown both before and after excluding outlier groups (e.g., 1-day curing). This consolidated summary facilitates a comprehensive understanding of which variables were statistically validated, how they were tested, and what level of influence they exert on compressive strength.

**Figure 22:**
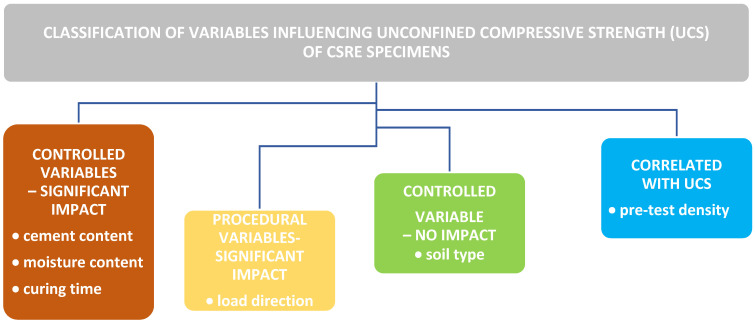
Classification of material and procedural variables according to their influence on UCS in CSRE specimens.

The normality of UCS distributions within each group of samples was verified using the Shapiro–Wilk test, confirming no significant deviation from normality (*p* > 0.05). This validation justified the use of parametric methods (ANOVA, Welch’s *t*-test) and strengthened the reliability of the statistical conclusions. A structured classification of the examined variables is presented in Figure 22. These findings applied specifically to the tested clayey silt (ML–CL) and to the moisture and cement ranges evaluated.

The drop-ball results presented in Table 5 showed a clear correspondence between moisture content and fragmentation behaviour. Mixtures below the optimum moisture content fragmented into numerous small particles, whereas mixtures prepared at the Proctor-identified optimum moisture produced only several larger fragments. Mixtures above the optimum remained largely intact. This behaviour confirmed that particle cohesion and fracture pattern were strongly moisture-dependent and aligned with the quantitative trend indicated by the modified Proctor compaction curve.

The central finding of this research is that soil preparation method (powdered soil vs. natural soil with clods) had no statistically significant effect on UCS. This result directly addresses the key methodological question and demonstrates that labor-intensive soil powdering—common in laboratory practice—does not provide measurable strength benefits when moisture and cement content are properly controlled. These findings challenged conventional laboratory practice by showing that soil powdering did not enhance UCS and should not be considered a necessary preparatory step.

By contrast, cement content, moisture content, and curing time were identified as the governing factors, each producing highly significant ANOVA results (*p* ≈ 0). Cement dosage and moisture define the chemical and physical conditions for hydration and bonding, while extended curing allows these processes to fully develop. Similar findings were reported in explainable artificial intelligence analyses, where cement content and moisture level were consistently identified as the most influential predictors of CSRE compressive strength [57]. These findings are consistent with previous studies and underline the need for strict control of these parameters in both laboratory testing and field applications.

Additional influences were also observed. Loading direction significantly affected UCS, underscoring the necessity of standardized testing procedures. Bulk density, although showing weak overall correlation with UCS, became a strong predictor of compressive strength once immature (1-day cured) specimens were excluded, highlighting its practical role as a proxy for compaction quality and mixture uniformity.

Moisture content proved particularly decisive. Results of the modified Proctor test demonstrated that even minor deviations from the optimum water content led to marked reductions in UCS, confirming the high sensitivity of CSRE to water variations. Complementary drop-ball tests provided outcomes consistent with NZS 4298:1998, confirming their utility as a rapid, empirical on-site method when supported by laboratory calibration. The correspondence between the drop-ball observations and the Proctor-based optimum moisture was qualitative rather than statistical, and therefore the method cannot be considered validated in a quantitative sense. The test nevertheless showed consistent qualitative behaviour across mixtures of different moisture contents, indicating its usefulness as a simple field indicator rather than a statistically supported laboratory method.

In summary, the classification in Figure 21 establishes cement content, moisture content, and curing time as the dominant factors; bulk density and loading direction as relevant secondary or procedural variables. From both scientific and practical perspectives, the findings emphasize that precise moisture control, curing, and binder dosage are far more critical for UCS performance than soil powdering. Eliminating unnecessary preprocessing improves laboratory efficiency and ensures closer alignment between research practice and real-world construction conditions.

## 6. Conclusions

The findings of this study demonstrate that the method of soil preparation—specifically, the use of powdered soil compared with natural, aggregate-retaining soil—does not exert a statistically significant effect on the unconfined compressive strength (UCS) of cement-stabilized rammed earth. The influence of soil type was negligible within the moisture and cement ranges examined in this study. This outcome indicates that soil pulverization, although common in laboratory practice, does not enhance mechanical performance when moisture content and cement dosage are properly controlled.

In contrast, cement content, moisture level, and curing time were identified as the primary determinants of UCS, each exhibiting a highly significant statistical influence. These parameters govern the chemical and physical conditions that regulate cement hydration and the development of interparticle bonding, thereby shaping the strength evolution of CSRE materials. Moisture proved particularly critical: even small deviations from the Proctor-derived optimum moisture content resulted in substantial reductions in UCS, underscoring the high sensitivity of CSRE mixtures to water content. Complementary drop-ball test results showed qualitative agreement with the Proctor curve, supporting its utility as a rapid field indicator of appropriate mixture preparation, albeit without replacing quantitative laboratory calibration.

Overall, the study highlights that the mechanical performance of cement-stabilized rammed earth is predominantly dictated by moisture control, binder proportion, and curing conditions, whereas soil pulverization and soil type have negligible influence within the investigated ranges. From a practical standpoint, removing unnecessary preprocessing steps can increase laboratory efficiency and yield results more representative of field conditions. These conclusions support the adoption of simplified yet robust testing procedures and provide guidance for future research and on-site applications involving CSRE materials.

## Figures and Tables

**Table 2:** Quantitative mineralogical composition of the natural soil sample.

Clay Minerals				Quartz (%)	Calcite (%)	Goethite (%)
Total (%)	Kaolinite (%)	Beidellite (%)	Illite (%)			
24.3	2.2	4.7	17.4	66.9	7	1.8

**Table 3:** Mineralogical composition of the modified natural and powdered soil.

Clay Minerals				Quartz and Others (%)	Calcite (%)	Goethite (%)
Total (%)	Kaolinite (%)	Beidellite (%)	Illite (%)			
8.1	0.73	1.57	5.8	83.3	8	0.6

**Table 4:** Rammed Earth Mixtures Designed for Compressive Strength Evaluation.

Sample ID	Earth Type: 1—Soil Powder; 0—Natural Soil	Cement Content (%)	Moisture Content (%)	Curing Time (Days)	Direction of Force: 1—Perpendicular; 0—Parallel	Number of Samples
1-s-C9-W9	1	9	9	28	1	6
4-w-C9-W9	0	9	9	28	1	3
4-w-C9-W11	0	9	11	28	1	3
4-w-C9-W13	0	9	13	28	1	3
3-w-C12-W9	0	12	9	28	1	3
3-w-C12-W9	0	12	9	28	1	3
3a-w-C12-W9	0	12	9	28	0	3
5a-w-C12-W9	0	12	9	1	1	4
7a-w-C7-W9	0	7	9	28	1	5

**Table 5:** Drop-test ball results for CSRE mixture with 9% cement addition.

**Drop-ball** **test results**	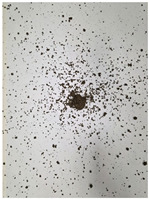	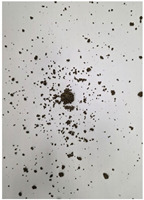	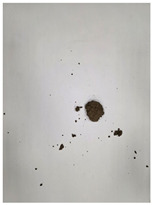	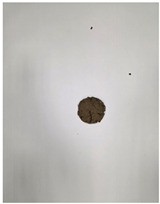	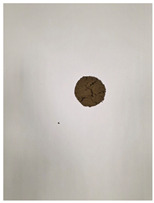
**Cracking** **behaviour**	*Ball shatters into many small angular fragments; brittle disintegration indicating mixture too dry.*	*Ball breaks into several medium fragments with visible sharp cracking planes; moisture still insufficient for cohesion.*	*Ball fractures into a few large compact fragments; cracking pattern consistent with optimum moisture.*	*Ball remains mostly cohesive with only 2–3 major cracks; onset of over-wetting with reduced brittleness.*	*Ball deforms plastically and remains largely intact; cohesive failure without distinct cracking planes, indicating excessive moisture.*
**Moisture** **content**	7%	8%	9%	10%	11%

**Table 6:** Unconfined Compressive Strength Results and Associated Variables for Laboratory-Tested Rammed Earth Specimens. Sample IDs followed the format: [Series]-[soil type]-C[cement content %]-W[moisture %]-sample number within series.

Sample ID	Earth Type: 1—Soil Powder; 0—Natural Soil	Cement Content (%)	Moisture Content (%)	Curing Time (days)	Pre-Test Density (kg/m^3^)	UCS (MPa)	Direction of Force: 1—Perpendicular; 0—Parallel
1-s-C9-W9-1	1	9	9	28	2113.34	14.69	1
1-s-C9-W9-2	1	9	9	28	2134.35	15.76	1
1-s-C9-W9-3	1	9	9	28	2144.56	14.21	1
1-s-C9-W9-4	1	9	9	28	2181.80	14.37	1
1-s-C9-W9-5	1	9	9	28	2198.86	14.65	1
1-s-C9-W9-6	1	9	9	28	2138.96	15.07	1
4-w-C9-W9-1	0	9	9	28	2204.06	15.01	1
4-w-C9-W9-2	0	9	9	28	2217.14	15.22	1
4-w-C9-W9-3	0	9	9	28	2207.71	15.54	1
4-w-C9-W11-1	0	9	11	28	2082.24	9.26	1
4-w-C9-W11-2	0	9	11	28	2023.78	9.62	1
4-w-C9-W11-3	0	9	11	28	2089.17	10.35	1
4-w-C9-W13-1	0	9	13	28	1989.31	6.87	1
4-w-C9-W13-2	0	9	13	28	1999.66	6.24	1
4-w-C9-W13-3	0	9	13	28	1999.06	6.65	1
3-w-C12-W9-1	0	12	9	28	2234.71	19.90	1
3-w-C12-W9-2	0	12	9	28	2271.67	21.40	1
3-w-C12-W9-3	0	12	9	28	2230.75	20.30	1
3a-w-C12-W9-1	0	12	9	28	2208.64	28.64	0
3a-w-C12-W9-2	0	12	9	28	2237.40	29.06	0
3a-w-C12-W9-3	0	12	9	28	2232.27	27.86	0
5a-w-C12-W9-1	0	12	9	1	2309.80	3.62	1
5a-w-C12-W9-2	0	12	9	1	2302.53	3.97	1
5a-w-C12-W9-3	0	12	9	1	2307.77	3.84	1
5a-w-C12-W9-4	0	12	9	1	2310.77	3.68	1
7a-w-C7-W9-1	0	7	9	28	2131.90	11.03	1
7a-w-C7-W9-2	0	7	9	28	2107.52	11.37	1
7a-w-C7-W9-3	0	7	9	28	2209.69	11.68	1
7a-w-C7-W9-4	0	7	9	28	2211.86	11.20	1
7a-w-C7-W9-5	0	7	9	28	2114.03	11.46	1

**Table 7:** Summary of Testing Protocols, Grouping Criteria, and Statistical Methods with results.

Variable	Tested Groups	Shapiro–Wilk	Levene’s Test	ANOVA	Welch’s Test	Pearson Correlation (with UCS)
**Cement** **content**	*Samples with equal* *moisture, curing time*, *load direction*	Passed	Passed *p* = 0.1136	Significant *p* = 0	N/A	N/A
**Moisture** **content**	*Samples with 9% cement, equal curing time* *and load direction*	Passed	Passed *p* = 0.55196	Significant *p* = 0	N/A	N/A
**Curing time**	*Samples with 12% cement, curing 1* *vs. 28 days*	Passed	Failed *p* = 0.0000	Significant *p* = 0.000016	Significant *p* = 0.000085	N/A
**Soil** **type**	*Samples with equal moisture and cement content*	Passed	Passed *p* = 0.2947	Not significant *p* = 0.2243	N/A	N/A
**Density**	*All samples/Excluding* *1-day samples/Excluding parallel loading direction*	N/A	N/A	N/A	N/A	0.200/0.758/0.853
**Loading direction**	*Samples with 12% cement, 28 days curing*	Passed	Passed *p* = 6067	Significant *p* = 0.0002	N/A	N/A

## Data Availability

The original contributions presented in the study are included in the article. Further inquiries can be directed to the corresponding authors.
